# A qualitative investigation on career barriers and career preparation behaviors of third culture kids in south Korean colleges

**DOI:** 10.3389/fpsyg.2024.1382179

**Published:** 2024-09-04

**Authors:** Kahyen Shin, Young-An Ra

**Affiliations:** ^1^Counseling and Counselor Education, Syracuse University, New York, NY, United States; ^2^Department of Psychotherapy, Myongji University, Seoul, Republic of Korea

**Keywords:** third culture kids, career barriers, career preparation behaviors, consensual qualitative research, Korean college students

## Abstract

**Introduction:**

The purpose of this study is to explore career experiences of South Korean Third Culture Kids (TCKs) and to examine their career barriers and career preparation behaviors.

**Methods:**

For these aims, eight South Korean TCKs were interviewed and Consensual Qualitative Research (CQR) was used to analyze the interview data.

**Results:**

As results, two domains, career barriers and career preparation behaviors were developed. For career barriers and career preparation behaviors of TCKs, five each core ideas were reported.

**Discussion:**

At the end of the study, we offered discussions and implications for higher education professionals and career counselors. The findings of the present study will contribute to the career development of TCK populations.

## Introduction

With globalization and technological advancements, the world is progressing towards increased global interconnectedness and growing multiculturalism on a national level (van der Zee et al., 2013). International mobility has surged, leading to a rise in children living cross-national, cross-cultural lives (Tan et al., 2021). This global movement exposes individuals to diverse cultures in various contexts, such as work, school, and personal life, fostering excellent cultural contact and interaction (Cheung, 2019). Children adopt international lifestyles for various reasons, including immigration, refuge, and expatriation (Tan et al., 2021). The growing prevalence of this phenomenon has sparked significant interest in Third Culture Kids (TCKs). Signifying a notable increase in TCK population, it is estimated that around 230 million individuals could identify themselves as TCKs (O’Bryan, 2022). Also called global nomads, TCKs act as cultural chameleons who adapt to their surroundings (Caselius and Suutari, 2023). Many articles argue that TCKs are often characterized by their open-mindedness and cultural sensitivity (Gerner et al., 1992; Selmer and Lam, 2004; van der Zee et al., 2013; Wisecarver, 2014; Cheung, 2019). Although exposure to diverse cultures has a positive impact on TCKs, it may also lead to the manifestation of certain characteristics, such as contending with ambiguity and uncertainty (Pollock and Van Reken, 2009). The demanding environment, marked by the challenge of establishing a stable sense of belonging, confusion in identity formation, and suppressed emotions arising from inevitable life losses, results in the display of superficial relationship patterns (Pollock and Van Reken, 2009). However, there are lack of past studies on this area which indicate the hardships that TCks are experiencing, therefore, more attention is necessary in this field of research as an attempt to bring attention to and scrutinize the difficulties faced by TCKs.

Particularly as TCKs complete college and transition into the workforce, they often experience heightened levels of anxiety and confusion as compared to individuals raised within a single culture (Pollock and Van Reken, 2009). According to Thurston-Gonzalez (2009), many TCKs report an international aspect in their lives is reflected in occupational preferences, leading to the conclusion that over half of the TCKs consider an international aspect when choosing their occupation. Some specified a particular location they wanted to work in, some aspired to work in an internationally-based occupation with frequent international moves, while others sought opportunities for job-related travel (Cheung, 2019). These TCKs also emphasized that the most significant influence their upbringing had on them was their chosen field of study in school (Wisecarver, 2014). Several TCKs reported that choosing such degrees was consciously linked to the desire to pursue a career abroad (Cottrell, 2002; Byttner, 2012). According to Cottrell (2002), TCKs consistently demonstrate high educational achievement, often leading to careers involving expertise, leadership, and independence. Pursuing an internationally oriented major or minor, many TCKs held degrees in international relations, anthropology, foreign language, and education. These patterns suggest that TCKs generally possess an overarching desire to contribute to the humanitarian field (Wisecarver, 2014), including professions in medicine, teaching, social work, clergy, or counseling (Byttner, 2012). As their overseas experiences grow and enrich them, TCKs may aspire to continue that inspiring experience by favoring a future internationally mobile career that is mobile, avoiding settling down in any one place (Gerner and Perry, 2000).

Although past literature has reported TCKs’ experiences upon their return to their home country on passport, there has been limited study explicitly focused on the career development of TCKs. As evidenced by prior literature, TCKs demonstrate a unique pattern in career development as compared to their domestic peers. Emphasizing the educational value of such studies, educators need to understand the behaviors and backgrounds of their multicultural students (Ra et al., 2024). Thus, this study specifically explores the career experiences and development of TCKs.

### Third culture kids: definition and characteristics

The concept of Third Culture Kids was initially introduced by Ruth Hill Useem and her husband, Useem et al. (1963). Useem (1973) defined Third Culture Kids(TCKs) as children of parents who work outside their passport countries and grow up in a third culture. TCKs spend a substantial portion of their developmental years, including their attendance at schools, in multiple places and multicultural environments due to their parents’ professions (Wisecarver, 2014). Hervey (2009) categorized four types of TCKs: (1) children of military personnel; (2) children of diplomats; (3) children of business professionals; and (4) children of missionaries or non-profit organization employees. According to Pollock and Van Reken (2009), TCKs are nurtured in a cross-cultural world, growing up in different countries and traveling back and forth between their passport and host cultures. Meanwhile, they actively involve themselves in the direct observation and analysis of diverse cultures, opting for experiential learning rather than relying solely on theoretical knowledge from books (Pollock and Van Reken, 2009).

TCKs differ from immigrant families, which generally come from a country and culture to which they belong (Tan et al., 2021). Immigrants follow a classic immigration pattern with a single move to a new country for permanent settlement, limiting additional mobility to trips within the new country or to the parent’s home country. In their new home country, immigrants are recognized as different from the dominant culture due to distinct appearances, speech, and habits. Both immigrants and host nationals expect immigrants to gradually acculturate and develop new roots of belongingness in the new country. In contrast, TCKs are perceived by their families, host country nationals, and themselves as belonging to their passport countries, even if they may not feel a belonging there due to limited birth or residency. Their mobility pattern involves temporarily moving abroad with the expectation of repatriating to their home or passport country, often living in one or several different host countries. Consequently, neither TCK families nor their host country nationals expect TCKs to root themselves and belong in the host country (Tan et al., 2021).

Pollock and Van Reken (2009) identified common experiences among TCKs, the anticipation of eventually returning to their parents’ home countries and a sense of identity connected to the values of the sponsoring agency, such as the ‘greater good’ (Bonebright, 2010). Having grown up amidst the blending boundaries of multiple cultures, TCKs are recognized for their heightened adaptability to thrive in ever-changing environments, coupled with the ability to appreciate differences without prejudice. Pollock and Van Reken (2009) indicated that exposure to diverse backgrounds and friendships with individuals from various cultures becomes a normal part of their experience. TCKs often use their cross-cultural encounters to understand and explain others’ behavior, contributing to their reduced tendency for prejudice. TCKs acquire the necessary skills in real-life situations during their adolescence, a phase when they are particularly open to learning from their cross-cultural encounters (Selmer and Lam, 2004). They exhibit proficiency in multiple languages and international perspectives, excellent crisis-coping skills, and a high degree of independence (Gerner and Perry, 2000). According to Thurston-Gonzalez (2009), TCKs typically have a more international mindset and consider it essential to incorporate an international aspect into their lives.

### South Korean TCKs in college

Starting college is frequently observed as the initial stage of adulthood, where adolescents form new behavior patterns and cognitive-emotional responses to address the challenges of their unfamiliar environment (Pratt et al., 2000; Tao et al., 2000). Given the potentially stressful nature of the shift to college, it is unsurprising that numerous students, particularly TCKs, may face challenges in this transition (Ra et al., 2024). TCKs typically exhibit greater flexibility, independence, expressiveness, and openness (Lam and Selmer, 2004; Lee, 2018). Moreover, they often demonstrate heightened objectivity, critical thinking, and resilience in their thought processes. However, these traits may lead to their exclusion in the school environment of a rigid society, as observed in South Korea (Kim and Yoo, 2008).

As noted by Ra et al. (2024), the South Korean education system stands distinct from others globally. It is highly systematic and leans towards conservatism, emphasizing uniformity in beliefs, values, and goals. The competitive nature of the environment, which creates a tense and apprehensive atmosphere for TCKs, is a primary contributor to the stress experienced by TCKs as they adapt to schools and post-secondary institutions (Iem and Shim, 2017). According to Iem and Shim (2017), challenges have been noted in psychological and socio-cultural adjustment, language acquisition, academic performance, and interpersonal relationships, signifying hurdles in academic adaptation. Upon their return to Korea, TCKs encountered difficulties aligning with the Korean curriculum, and they struggle to quickly adapt, to standard classes within the country, resulting in significant stress (Lee, 2018). The additional hurdles arising from differences in teaching and learning methods, coupled with academic stress associated with exams, were reported to contribute to their psychological strain. On the relational aspect, TCKs tend to have good manners and honesty in their friendship. TCKs who are able to speak English well were perceived as socially attractive by domestic students (Kwon, 2018). However, the results also indicate the challenges associated with collectivist culture in Korea, given that TCKs often become accustomed to individualistic culture country (Kim and Kim, 2014). As a result, TCKs experience both academic stress and cultural adjustment stress simultaneously upon returning to their home country. These results align with the research findings on the friendships of TCKs in the United States (Choi et al., 2013), and similar challenges in readjustment after repatriation have been reported in Western studies.

### Career experiences of TCKs

Navigating the path to an adult career involves meticulous planning, decision-making, and building the confidence to attain career objectives—a fundamental responsibility during adolescence and early adulthood (Stringer et al., 2011). For TCKs, careers appear to hold significance and value beyond mere survival. A career is perceived as a tool for actualizing the external and internal values that one considers important (Kim et al., 2018). These characteristics of TCKs may contribute to the creation of career barriers, as it takes them a longer time to develop a firm career path based on reflection of their international and multicultural identities. According to a previous study (Kim et al., 2018), career barriers for TCKs were identified as career changes/uncertainty, cultural and language difficulties, interpersonal relationships, lack of information, and financial difficulties. These reflects the challenges experienced in career preparation behaviors. TCKs grapple with uncertainty and anxiety about their identity upon returning home, a concern that persists into the future (Iem and Shim, 2017). Additionally, the career barriers faced in junior and senior years in college, occurring approximately 1–2 years after repatriation, appear to exacerbate existing weak identity confusion or lead to a process of reconfirming negative self-identity. Individuals may intensify the confusion or abandon domestic settlement, feeling detached and unable to settle anywhere (Kim et al., 2018). Despite being college students expected to have independence, TCKs still struggle with identity formation due to the characteristic of delayed adolescence (Pollock and Van Reken, 2009). These traits of TCKs appear to result in passive approaches and delayed career preparation behaviors.

Given that career preparation stands out as a key developmental task for college students (Arnett, 2000), previous studies have proposed that engaging in targeted career preparation enhances the probability of individuals selecting their desired careers (Ra and Kim, 2023). Consequently, career preparation behavior not only plays a crucial role in enhancing individuals’ quality of life but also emerges as a significant aspect in the realms of education and counseling (Park et al., 2021). Career preparation behavior encompasses (a) effective self-information gathering activities, including one’s abilities, aptitude, interests, and personality, along with exploring information about the desired work environment, employment outlook, and job requirements; (b) preparatory activities aimed at obtaining the necessary materials, methods, tools, or pathways to achieve career goals, involving actions like acquiring equipment, books, and materials for licenses/certificates; and (c) efforts directed towards actualizing specific career goals, such as investing time and energy in utilizing acquired tools and resources, exemplified by activities like studying for a certificate course (Kim, 1997).

In the realm of career preparation behavior, disparities exist in the definition and significance of a career when comparing TCKs and domestic students of the same age group (Kim et al., 2018). These differences stem from the substantial impact of distinct developmental processes, growth cultures, and backgrounds within these two groups. Domestic students tend to perceive career choices as synonymous with employment, prioritizing practical factors such as the realistic prospects of securing a job over preferences for aptitude or interests in the career selection process (Iem and Shim, 2017). In addition, domestic students compare and select employment opportunities based on factors like high income, welfare benefits, and potential for advancement, tailoring their skills to fit the chosen industry. The domestic student population generally favors positions in government-affiliated enterprises or large corporations that provide economic stability and future satisfaction. In contrast, TCKs aspire to pursue professions where they can leverage their unique expertise, leadership skills, and independence, influenced significantly by diverse cultural and socio-economic experiences during developmental period (Cottrell, 2002). TCKs tend to embrace high mobility (Selmer and Lam, 2004), favoring organizations that acknowledge individual independence and differences and which maintain a generous and inclusive culture (Bonebright, 2010). Therefore, the career preparation behavior of TCKs can vary, reflecting potential distinctions in their career priorities and values compared to those of domestic students. TCKs might encounter challenges during their career preparation due to readjustment and new identity formation, along with career decision-making. According to the study of Kim et al. (2018) expressed a sense of regret and frustration for their delayed career preparation which caused them career stress during their senior years.

In conclusion, this study highlights the intricate challenges faced by TCKs in their career development. TCKs experience heightened uncertainty and anxiety about their identity upon returning home, further complicating their career preparation behavior as well we their career path. Many previous studies (Ra and Kim, 2023; Ra et al., 2024) which explore TCKs unique experiences have mentioned that qualitative research is necessary for TCK research to understand their life more deeply. The study thus underscores the importance of recognizing these unique challenges that they are experiencing and advocates for targeted support to facilitate the successful career development of TCKs employing a qualitative research method.

## Methodology

### Participants

This study was conducted with eight samples of TCK college students in South Korea. Hill et al. (2005) reported in their study that at least eight to fifteen participants are an adequate sample which researchers can confidently confirm that the findings are not merely representing one or two people, but can be applied more generally. All of the participants are college students in Gyeongsang province, South Korea. The participants’ age ranges from 21 to 26 and five people are men and three people are women. Their length of the stay in abroad ranges from 3 to 16 years.

### Instruments

According to Hill et al. (2005), semi structured interview is highly recommended because it creates comfortable atmosphere for the participants to freely answer in an unstructured form and it also helps the interviewer to get desired information by leading the participants when they are straying from the topic. Participants completed a one-page informed consent form. This form asked them for the following information: age, gender, major, 2nd culture country, length of stay in 2nd culture county, what school they attended in the 2nd culture country, and availability for the interview. Moreover, the informed consent form contains an agreement of interview recording.

The participants voluntarily signed up the consent form, and by signing the sheet, the participants’ confidentiality was firmly guaranteed. Once the participants agreed to sign this form we started the interview. Then the interview followed a semi structured format which enabled the data to emerge from the participants freely. The interview protocol was developed by researchers who explored previous studies (Gerner et al., 1992; Selmer and Lam, 2004; Thurston-Gonzalez, 2009; Byttner, 2012; van der Zee et al., 2013; Wisecarver, 2014; Cheung, 2019) on this related topic. The semi-structured interview protocol included several questions such as “What are your career experiences?” “Were there any barriers that hindered your career?,” “What factors helped you to prepare and develop your career?.” As the interview are implemented, the researchers asked more questions according to the flow of the interview.

### Procedure

The data was collected by the following three steps: (a) conducting the interviews, (b) Taking notes about the participants’ impressions during the interviews, and (c) transcribing the interviews. Two researchers conducted the interviews and each interviewer was in charge of four participants. By using two interviewers, the interviews reduced the possible effects of interviewer style and bias. The interviews were designed to proceed last about 40–50 min in length. Each interview was followed by an explanation about what the interview was about, where and how it will be used, and also allowed the participants to express any curiosities and opinions they might have. The interviewers practiced the interview in advance to ensure whether they were delivering the questions clearly and convincingly.

During each interview, the interviewer took notes on how the participants reacted and the impressions the interviewer about the participants. Then transcribers listened to the recordings in a quiet place and kept everything the heard confidential. After the transcripts were typed, a researcher went through the transcript against the recorded interview to ensure accuracy and also edited errors which could be problematic when analyzing the data. Moreover, the researcher deleted each participants’ proper name to maintain confidentiality.

### Data analysis

This study employed consensual qualitative research (CQR), which is a qualitative method that can be used to explore individuals’ inner experiences, attitudes, and beliefs, all of which are not readily observable. CQR is considered as an ideal method because researchers can interview people to find out in-depth information that cannot easily be found using traditional experimental and quantitative methods in education and psychotherapy research. After the data were collected, a data analysis was conducted in five steps: (a) developing domains, (b) constructing core ideas (c) examination of the domains and core ideas with the auditor (d) cross analysis and (e) stability check. This study consists of two members of a core research team and one auditor. The members of the core research team are studying counseling psychology in the University. The research team and the auditor met for an average of 2 h weekly for about 3 months. On the days which they did not hold a meeting, the research team independently analyzed the data into domains, core ideas, and contents and then cross analyzed each other’s data. During meetings two researchers and the auditor converged the findings to a consensus. Finally, all researchers and the auditor found the data stable by checking all of the domains, core ideas and contents. Lastly, the researchers classified the core ideas and contents as general for all cases, typical for more than half cases, and variant for less than half cases referring to the definitions in Hill et al. (2005). Finally, research created a table for the results to explain with details according to Hill et al. (2005)’s presentation.

## Results

Results of the data analysis showed the two domains: (a) career barriers and (b) career preparation behaviors. A more detailed description about each domain is provided in Table 1. The core ideas and contents that emerged for each domain are also explained. The research team classified domains, core ideas and contents as general, typical, and variant in accordance with Hill et al. (2005)’s study. All domains were considered general, while a great part of core ideas were considered general and typical. Lastly, the majority of the contents were regarded as typical and variant, and a small number was regarded as general.

**Table:** 

Domain	Core idea	Content	Frequency
Career Barriers	Language	Not fluent in Korean	Typical
		Jobs where English can be used are only considered.	Typical
	Cultural Differences	Lack of understanding of Korean culture	Typical
	Interpersonal Relationships	Hard to find a person to consult	Typical
		Absence of mentor or role model	Variant
	Lack of Information	Do not know the Korean job market	Typical
		Hard to find employment information	Typical
	Financial Difficulties	Lack of financial resources	Variant
Career Preparation Behaviors	Academic Performance	Improving GPA	General
	Networking	Professors	Typical
		Alumni	Typical
	Gaining information	Study meeting with friends	Typical
		Career Counseling	Variant
		Using internet	General
	Occupation requirements	Work Activities	Variant
		Internships	Typical
	Experience requirements	Licenses	Variant
		Certificates	Variant

**Table 1:** Participant information.

	Age	Gender	Major	2nd culture country
1	24	Female	Psychology	India
2	24	Male	English	U.S.A
3	23	Male	Management	China
4	26	Male	Law	Phillipines
5	22	Female	English	U.S.A
6	24	Male	Psychology	Vietnam
7	21	Female	English	Thailand
8	23	Male	Psychology	China

### Domain 1: career barriers

The first domain was career barriers. The career barriers domain was general. Within this domain, there were five core ideas, and they were all typical. The five core ideas were language, cultural differences, interpersonal relationships, lack of information, and financial difficulties. Each core idea and content are discussed below.

#### Language

The core idea, language includes the contents which is Korean fluency. Participants talked about how Korean fluency is important in employment in Korea.

“Not being able to speak Korean well can be an obstacle to getting a job. These days, there are many jobs that want people who are good at both Korean and English.” < *Participant 2*>.

#### Cultural differences

The core idea, cultural differences refers to lack of understanding of Korean culture as well as Korean organizational cultures. First, participants illustrate that as TCKs, insufficient understanding about Korean culture is not helpful to the pursuit of a career in South Korea.

“Because I am not familiar with Korean culture. When deciding on a career path, not knowing much about Korean culture has a negative impact on my employment.” < *Participant 1*>.

Participants also reported that lack of knowledge about Korean organizational culture can be a barrier to their career development. They also mentioned that they may feel afraid of getting a job without this knowledge.

“Sometimes, I am afraid of getting a job because I do not understand much about Korean organizational culture. Later, I may end up regretting my career decision.” < *Participant 6*>.

#### Interpersonal relationships

The core idea, interpersonal relationship as a career barrier includes two contents which are difficulties to find a person to consult and absence of mentor or role model. Over half of the participants noted that it is hard to find a person they can consult with about their career.

“I need someone who understands my unique situation. But it’s hard to find someone like that. Even if I wanted to discuss my career, I have nowhere to go for advice.” < *Participant 5*>.

Some participants also acknowledged the power of a mentor or a role model. Participants reported the importance of mentoring or modeling.

“It’s hard to find a mentor. If there were similar people around me, I would watch and learn from them.” < *Participant 1*>.

#### Lack of information

The core idea, lack of information includes two contents which are not enough understanding about Korean job market and difficulties to find the information about target employment. TCK students reported that the fact that they do not have enough information about the Korean labor market when compared with domestic college students can be a significant career barriers for them.

“The fact that I know less about the job market in South Korea than my Korean friends could mean that I am a less competitive person.” < *Participant 2*>.

They also mentioned that sometime it is sometimes difficult to find proper information about the career that they want. They said it seems that how much information they have is important for their career paths.

“But it’s really hard to find information. It seems completely different from the way I search for information before. I want to find information, but I often end up wasting my time.” < *Participant 4*>.

#### Financial difficulties

TCK students also mentioned that they considered financial difficulties as their career barriers. They said that they always think about their financial situation when they decided their career.

“My parents have helped me so much so far that I cannot help but think about the financial situation. Because I have to get a job right after college graduation, I cannot think about my career for such a long time, because of money” < *Participant 3*>.

### Domain 2: career preparation behaviors

The second domain was career preparation behaviors. Within the career preparation domain, there were also five core ideas. The contents were academic performance, networking, gaining information, occupational requirements, and experience requirements. Each core idea and content are discussed below.

#### Academic performance

The first content of career preparation behaviors was academic performance. Almost all of the participants reported that their academic success is important for their career. To prepare for their career, it seems that they have tried to improve their GPA in college.

“I heard that when applying for a job, employers usually consider significantly my college GPA. Although I have little experience in Korea, I think it will be advantageous to have good grades at college, so I always pay attention to my GPA.” < *Participant 7*>.

#### Networking

The core idea, networking is comprised of two contents, professors and alumni. One participant reported to the interviewer that he got so much help from professors whenever he encountered difficult situations in every aspect. The first thing he did to create his network was visiting professors.

“The professors helped me a lot. They introduced me to people I needed to decide on my career path, and also connected me with students in similar situations.” < *Participant 1*>.

Other participants also emphasized that networking is important. They mentioned that they have created their networks through the seniors who have graduated from the same major at college.

“I met a lot of seniors from my major and expanded my network. When you meet seniors, I somehow obtain a lot of information and connections for my career.” < *Participant 8*>.

#### Gaining information

As for their career preparation behaviors, participants reported that gaining information relating career is crucial. This core idea included three contents which are study with friends, career counseling center, and on-line websites. Some students mentioned that they could get a lot of information relating to their career through study meetings with friends.

“There are many friends in this university who are in special situations like us, I mean TCKs. Those friends get together and talk together. We also share the information that we already have and look for them together. I feel like there is a need for doing it right now, so we get along well.” < *Participant 8* >.

The career counseling center on campus was another resources for them to get career information. They mentioned that counseling sessions that they had at the career counseling center were very helpful.

“If we go to the career development center, the professionals provide as much career information as possible. I can also receive career counseling. I had great help from the career counseling. Every time the university offers many career-related events, such as mentoring program or career camps, these programs are very helpful.” < *Participant 7*>.

Lastly, the participants also reported that they mostly obtain information through the websites. These websites included organizations’ webpages and career or job-related websites.

“I usually find information through the internet. Visiting admissions of graduate school or organizations’ webpages to find out what I need to do.” < *Participant 3*>.

#### Occupation requirements

The core idea, occupational requirements is comprised of work activities and internships. Many of the participants experienced work activities related their target occupations and internships.

“I think it’s important to prepare my career by working through something like an internship. It is also important to show that I have done a lot of related work before graduating from college.” < *Participant 5*>.

#### Experience requirements

For their career preparation behaviors, participants mentioned about career experience. This experience requirements included two contents, licenses, and certificates. They emphasized the importance of achieving licenses or certificates for their career.

“I need to obtain a license or a certification. This is what objectively shows my abilities in Korea. When students reach their senior year, they put a lot of effort into obtaining these qualifications to prove their capabilities.” < *Participant 4*>.

## Discussion

This study aimed to explore the career development of TCKs in South Korea. To accomplish this goal, we examined the career development experiences of TCKs during their college years. The collected data were categorized into two domains: career barriers and career preparation behaviors. In this section, we provide detailed insights into each domain and highlight the core ideas that emerged from the research data.

The first core idea within the domain of career barrier was language. TCKs often attend international or local schools where they acquire proficiency in foreign languages (Kim et al., 2018). According to Kim et al. 83.7% of Korean TCKs are fluent in English, while the rest are proficient in other foreign languages. Cheung (2019) reveals that 37% of the TCKs are proficient in four languages, 46% speaking three languages, and 17% claim proficiency in two languages or fewer. This multilingual ability of TCKs can be viewed as a competitive advantage as compared to domestic students (Kim et al., 2018; Ra et al., 2024). However, despite their multilingual skills, TCKs often face have challenges due to their limited fluency in advanced levels of the Korean language (Iem and Shim, 2017). Since workplaces in Korea often require a higher level of proficiency beyond conversational Korean, our participants reported feeling less competent in the job market. This aligns with earlier literature, which identifies proficiency in the Korean language as a significant factor impeding smooth transitions for TCKs (Kim et al., 2015).

Another core idea, cultural differences, was identified as a career barrier for TCKs. South Korea is a predominantly a homogeneous society that discourages non-conformity, creating an environment that induces uneasiness and anxiety among TCKs (Lee et al., 2022). Our participants also expressed unfamiliarity and anxiety regarding adapting to Korean organizational cultures. This result aligns with the study of Kim et al. (2018), where TCKs reported understanding Korean culture, characterized as collectivistic or competitive, as a career barrier. Additionally, our findings are consistent with reports of TCKs in college culture, as they often feel a sense of detachment due to cultural differences (Gibson, 2004; Pollock and Van Reken, 2009). Kim et al. (2018) noted that the adaptation period to a new culture contributes to TCKs’ delayed career development. According to our participants, TCKs reported that their lack of knowledge of Korean culture has a negative impact on their employment prospects in Korea. This cultural difference can limit TCKs’ options, aligning with previous literature suggesting TCKs plan to return overseas because they feel restricted when working in their home country due to cultural differences (Ra et al., 2019).

Interpersonal relationship represent one of the core ideas of career barrier. The lack of interpersonal relationship reflects a significant loss, such as the absence of someone to consult (i.e., mentor or role model). According to Ra et al. (2024), TCKs often seek to share their experiences with someone who has undergone similar cross-cultural experiences. In the selection of role model, empirical evidence strongly supports the role of perceptions of similarity as a variable (Gibson, 2004). Gibson noted that role models with a similar background can offer opportunities for learning, motivation, and self-definition. In this context, TCKs often face challenges in finding someone who is similar to them, and our participants’ responses align with previous literature. The presence of a mentor or role model signifies not only the importance of interpersonal relationships but also the significance of the source of career information.

Another core idea of career barrier, lack of information comprises two contents: information about the Korean job market and employment. While domestic students typically prepare for their career paths in secondary school (Lee and Kim, 2004; Ra et al., 2019), TCK college students, attending schools out of Korea, may not have access to as much career information and, therefore, feel less prepared compared to domestic students. This echoes the findings of our study, indicating that TCK participants experience career barriers due to insufficient career information about the Korean job market and employment.

Financial difficulties were identified as another core idea of career barrier. Despite a lack of research attention on the financial status of TCKs, their financial situations can vary based on their parents’ availability of support, considering the diversity of their parents’ professions (e.g., military personnel, diplomats, business professionals, and missionaries). In our study, one TCK participants reported the need to secure a job immediately after college due to lack of financial support from one’s family. Toyokawa and Dewald (2020) revealed the negative impact of a lack of time and financial support on career decisiveness. Consequently, the financial difficulties faced by TCKs can lead to hasty career decisions, negatively impacting their overall career development. Consistent with our findings, Kim et al. (2018) also reported that financial difficulties can result in delayed employment, coupled with lack of financial support for professional development such as license/certificate preparation, contributing to an overall delay in career development (Lin et al., 2023).

Career preparation behavior, the second domain of our results, consists of five core ideas. The first of these is academic performance. The TCK participants of our study reported focusing on maintaining a good GPA because they were uncertain about what to prepare for in their careers. Our findings align with the study of Kim et al. (2018), where TCKs expressed studying for their majors because they felt frustrated in the career preparation process—finding information was challenging, and they were unaware of specific career preparation actions. While domestic students also prioritize their GPAs as part of their career preparation behaviors (Lee and Kim, 2004; Ye et al., 2023, 2024), the emphasis placed by TCKs on improving their GPAs in college reflects a limitation in pursuing other career preparation behaviors.

The second core idea within this domain was networking, which consists of two contents: professors and alumni. Our findings include a report from a TCK participant who visited professors seeking assistance. He mentioned that his professor facilitated connections with helpful individuals and other students facing similar situations. Some participants also reported that their seniors were instrumental in expanding their career networks. This proactive approach to with other literature (Ra et al., 2019, 2024), exemplifying their ability to cope with cultural difficulties. Additionally, they actively engaged in exchanges with professors and peers as a strategy to navigate diverse academic situations. Therefore, our findings suggests that TCKs are notable for their proactive approach in extending their career networking.

Gathering information is another core idea within the domain of career preparation behavior. This includes contents such as study meeting with friends, career counseling and using the internet. Similar to the networking result, our findings about gathering information highlight how TCKs actively seek information while interacting with peers who share a similar background to receive support. This aligns with the research conducted by Ra et al. (2024), where TCKs frequently express a desire to share their experiences with individuals who can comprehend their perspectives. Moreover, the other contents, gathering information from websites and career counseling, emerged as a common career preparation behavior for both TCKs and domestic students. This is supported by reports from domestic students who benefit from career counseling and mentoring programs on campus (Lee and Kim, 2004; Ye et al., 2024). Our study also indicates that TCKs acquire information through job websites and career counseling.

The last core ideas were occupational requirements and experience requirements. They includes contents such as work activities, internships, certificates and licenses. In previous study by Lee and Kim (2004), Korean domestic students were reported to prepare for language proficiency exams (e.g., TOEIC, TOEFL, HSK, or JLPT). Based on previous literature regarding TCKs’ common proficiency in multiple languages (Gerner and Perry, 2000; Kim et al., 2018; Cheung, 2019), TCKs who already possess multilingual language abilities might require less preparation for language proficiency exams. As for the other requirement contents, many domestic students prepare for their careers by undertaking internships and obtaining licenses and certificates during their college years (Lee and Kim, 2004; Ye et al., 2023, 2024). TCKs in our study also reported eagerness to explore internship opportunities and pass certificate exams as they approach senior year, which is considered normal career preparation behavior in South Korea.

Taken together, TCKs encounter unique challenges, including a lack of domestic language proficiency, cultural differences, and limited access to interpersonal relationships and career information. Despite these barriers, TCKs exhibit proactive approaches in networking, gathering information, and meeting occupational and experience requirements. These findings underscore the significance of understanding TCKs’ distinct perspectives and needs in navigating their career development. It is evident that TCK’s multilingual abilities and international exposure contribute to their competitiveness, yet challenges persist in integrating with the local culture and securing essential resources.

This study encourages a nuanced understanding of TCKs’ career experiences, emphasizing the need for tailored support and interventions to enhance their career development in colleges. The findings of the present study suggest universities need to understand the unique backgrounds of TCK students and help them by developing many related counseling programs. Academic counseling may be needed for these students mainly focused on their learning, including understanding the courses and course systems they were majoring in, setting and realizing academic as well as career goals, and coping with difficulties (Wang et al., 2024; Ye et al., 2024). Moreover, career counseling programs in college would be able to help them adjust to life on campus as well as gain opportunities to their career paths (Datu and Buenconsejo, 2021; Ye et al., 2023, 2024). Higher education professionals could thus help those students reduce the difficulties faced by TCKs in post-secondary education, while helping them authentically experience their transition process into their next career steps (Lin et al., 2023; Liu et al., 2023).

There is a limitation in the present study. When selecting participants, we randomly selected the TCK students. Also, participants were college students from one university in South Korea. Thus, thus this can make generalizing the results of the study difficult.

## Conclusion

The present study was aim to explore the career experiences of TCK college students in South Korea. In the results of the study, career barriers and career preparation behaviors were reported as main domains of their career experiences and five each core ideas were reported as variables related their career. We believe that the results of the present study will be beneficial for many career counseling and student affairs professionals to effectively help TCKs populations on college campuses. Moreover, we believe that the results of this study will be useful for the TCKs themselves to help them manage their career difficulties in college and prepare for their futures.

## Data availability statement

The raw data supporting the conclusions of this article will be made available by the authors, without undue reservation.

## Ethics statement

Ethical review and approval was not required for the study on human participants in accordance with the local legislation and institutional requirements. The participants provided their written informed consent to participate in this study.

## Author contributions

KS: Writing – original draft, Writing – review & editing. Y-AR: Conceptualization, Methodology, Writing – original draft, Writing – review & editing.
